# Setting the agenda for nurse leadership in India: what is missing

**DOI:** 10.1186/s12939-018-0814-0

**Published:** 2018-07-09

**Authors:** Joe Varghese, Anneline Blankenhorn, Prasanna Saligram, John Porter, Kabir Sheikh

**Affiliations:** 1 Public Health Foundation of India, Delhi NCR, Plot No. 47, Sector 44, Institutional Area, Gurgaon, 122002 India; 2 Independent Consultant, Geneva, Switzerland; 3 London School of Hygiene & Tropical Medicine, Keppel Street, London, WC1E 7HT UK; 4 Nossal Institute of Global Health, University of Melbourne, Melbourne, Australia

## Abstract

**Background:**

Current policy priorities to strengthen the nursing sector in India have focused on increasing the number of nurses in the health system. However, the nursing sector is afflicted by other, significant problems including the low status of nurses in the hierarchy of health care professionals, low salaries, and out-dated systems of professional governance, all affecting nurses’ leadership potential and ability to perform. Stronger nurse leadership has the potential to support the achievement of health system goals, especially for strengthening of primary health care, which has been recognised and addressed in several other country contexts. This research study explores the process of policy agenda-setting for nurse leadership in India, and aims to identify the structural and systemic constraints in setting the agenda for policy reforms on the issue.

**Methods:**

Our methods included policy document review and expert interviews. We identified policy reforms proposed by different government appointed committees on issues concerning nurses’ leadership and its progress. Experts’ accounts were used to understand lack of progress in several nursing reform proposals and analysed using deductive thematic analysis for ‘legitimacy’, ‘feasibility’ and ‘support’, in line with Hall’s agenda setting model.

**Results:**

The absence of quantifiable evidence on the nurse leadership crisis and treatment of nursing reforms as a ‘second class’ issue were found to negatively influence perceptions of the legitimacy of nurse leadership reform. Feasibility is affected by the lack of representation of nurses in key positions and the absence of a nurse-specific institution, which is seen as essential for creating visibility of the issues facing the profession, their processing and planning for policy solutions. Finally, participants noted the lack of strong support from nurses themselves for these policy reforms, which they attributed to social disempowerment, and lack of professional autonomy.

**Conclusions:**

The study emphasises that the nursing empowerment needs institutional reforms to facilitate nurse’s distributed leadership across the health system and to enable their collective advocacy that questions the status quo and the structures that uphold it.

## Background

Nurses form the backbone of India’s health system representing 30.5% of all health workers in India [1]. Nevertheless, the inability to train, retain and deploy an adequate number of qualified nurses has been recognized by experts as one of the greatest challenges for achieving health system effectiveness [2, 3]. It is estimated that India needs an additional 2.4 million nurses to reach their optimal number in the health system [4, 5]. Recent initiatives have attempted to redress the nurses’ shortages in health care delivery and tried to correct imbalances in their geographical distribution [6].

Nevertheless, the lack of numbers is not the only problem faced by the Indian nursing. Their role in decision making, both in the clinical and public health domains is not recognised in India. Several experts have documented the state of affairs of nursing in the public and private sector and the official apathy towards them. They have described how status hierarchy among various cadres of health professionals which positions nurses at a lower place compared to medical professionals and the nurses’ dominant gender identity within the socio-cultural contexts constrain their ability to take up leadership positions in their own professional realm as well as in health sector [7, 8]. Nair and Prescott have argued that nurses’ compromised professional and social acceptance has direct repercussions on their performance and contribution to health system [9]. The low position of nurses in the hierarchy of health care professionals, poor working conditions, low salary, and out-dated personnel norms, all operate in vicious cycles to create compromised professional position of nurses. Skill development and career progression opportunities for nurses in India are meagre [8]. Abjuration of several nursing leadership positions at the district and state level in the public sector has been reported in a previous study [10]. Likewise, the experts who have commented on a major strike of the nurses working in private sector in the national capital had identified exploitative working conditions akin to bonded labour as the reason for their strike [9].

The current situation of nursing in India warrants several policy reforms to counter the adverse service and social conditions in order to facilitate their overall professional contribution as a valuable human resource. The situation of nurses has special significance for equitable health services in developing country contexts such as in India as most of them are placed as the frontline health workers in remote and difficult locations and play key role in addressing various social determinants of health. Several official committees appointed by government to look into nursing issues in the past have recommended various reform proposals. These recommendations vary from increasing the number of nurses in the health system to establishment of key positions for nurses in the higher bureaucracy. Nevertheless, the recent reforms have only focused on recruiting and deploying more nurses and the leadership challenges faced by nurses are generally overlooked.

The agenda of building nurse leadership occupies a central position in the WHO’s strategic directions for nursing and midwifery globally, in recognition of their potential to act as “agents of change” within health systems [11]. The 1987 WHO report on nursing promotes a vision of nurse leadership which is about creation of ‘empowered nurses’ who mobilize, influence and collaborate at multiple levels [12]. Previous studies on nursing have established the link between strengthening nursing leadership and better health system performance [13–15]. However, nurses’ leadership potential is negatively impacted when their status among the professional hierarchy are compromised compared to medical and other health professionals [16, 17].

Most literature on nurse leadership conceptualise leadership as individual ability to influence others towards achievement of relevant organizational goals [13, 14]. In this article, we examine nursing leadership as a means to strengthen the health system, by building structures that facilitate and support the leadership potential of every nurse in the system in order to achieve health system goals [18]. This concept of ‘distributed leadership’ is a shift from focus on the agency of individual leaders to the characteristics and design of the systems that facilitate leadership culture [19, 20]. The focus of this paper is on the policy reforms that are required to address the structural and systemic change that could facilitate nurses’ distributed leadership and enhance their roles as “agents of change in the health system”.

This study explored the process of agenda-setting for policies that could facilitate distributed nurse leadership in India, in public and private sector and in clinical and public health field, with the intention of understanding what factors are preventing action to strengthen the nurse leadership in India. Analysing the ‘agenda setting’ process is a way of understanding which issues, under what contexts gain policy attention. A key focus of such research is the attention on the process through which new ideas or policies may or may not be accepted within a political and policy system [21]. Therefore, research on agenda setting may also help in understanding the dynamics of status quo in policy process and explore potential pathways through which change can be facilitated.

## Methods

In order to understand the policy propositions for addressing nurses’ leadership crisis, we reviewed various key official documents at the national level and their prescriptions regarding nursing administrations, education and deployment. Two key documents included in this review are a report of the High Power Committee set up by the government of India that recommend several nursing reforms and a review report of status of nursing in five states undertaken by the National Health System Resource Centre, Ministry of Health and Family Welfare and ANSWERS [22, 23]. The other documents are Bhore committee report of 1946, the Indian Nursing Council Act of 1947 and its three amendments, the code of ethics and professional conduct prescribed by the Indian Nursing Council, the professional conduct etiquettes by Trained Nurses Association of India, Chadah committee report of 1963, National Health Policies of 1982 and 2002 and Clinical Establishment Act of 2010. The review of these documents helped to identify the reform proposals, status of their implementation and various challenges with respect to the agenda of strengthening nursing leadership.

In addition, nine (09) interviews took place (between 5 and 24 August 2013) in New Delhi, Andhra Pradesh and Kerala with experts who were identified according to their interest in nursing governance and the extent to which they have information and knowledge on decision-making and implementation of policies dealing with nurse leadership. Though three interviews were carried out at the state level, all interviewees had expertise on nurse leadership issues at the national level. Except for three, all the other participants were qualified nurses. The Table 1 describes the characteristics of Key Informants interviewed in the study.

**Table 1:** Characteristics of interviewees

Main characteristics of interviewees	Number of Interviewees	Code
Senior government officials dealing with nursing administration	2	IA, IB
Office bearers of two national level nurses’ associations	2	IC, IG
Researchers on nursing issues in the country	2	ID, IE^a^
Representatives of two resource organizations supporting nursing reforms	2	IF^a^, IH^a^
Leading nurse educator in the country	1	II

^a^Non-nurse participants

Though this study was based on limited number of expert interviews, it was felt that saturation was reached with each informant identifying similar barriers to nurse leadership and solutions to these.

A topic guide was developed to help conversations with informants and questions were formulated with the aim of obtaining relevant, complete and contextualized information on nurse leadership and on appropriate policy solutions that address nurses’ leadership crisis in India. The interviews were held in August of 2013 and started by exploring the context of nurse leadership in India and structural and systemic constraints in setting the agenda for policy reforms on the issue.

All the interviews were in English and handwritten notes were taken at the time of interview and later transcribed into text format. Data were thematically organized and classified according to ‘legitimacy’, ‘feasibility’ and ‘support’, in line with Hall’s agenda setting model [24]. The model stipulates that a policy issue needs to fulfil the criteria of legitimacy, feasibility and support in order to be included in the policy agenda. Legitimacy is conceptualised as the “*characteristic of issues which governments believe they should be concerned with and in which they have a right or even obligation to intervene*”. Feasibility is about implementation potential, which is dependent upon i) technical and theoretical knowledge; ii) financial resources and human capital; iii) administrative capability and infrastructure. ‘Support’ denotes the level of public support for government in relation to the issue.

Ethical clearance was obtained from the Ethics Committee of London School of Hygiene and Tropical Medicine, the institutions of two of the authors. Consent was obtained from the study participants interviewed, after providing them with information regarding the study’s aims and objectives. Consent forms ensured their confidentiality and anonymity, and codes were assigned to protect their identity.

## Findings

Drawing on the document review, we first clarify the structure of nursing institutions in India. Section B then presents main policy reform proposals for advancing nurse leadership extracted from our review of the key documents and the status of these reforms at the time of the study. Based on the core objectives of these reform proposals, we group them under two headings; policies proposed for creating social mobility and policies for strengthening nursing institutions. Under section C, based on expert interviews which reflected on contextualised assessment of the policy process concerning nurses’ leadership reforms in India, we outline our findings about agenda setting using Hall’s agenda setting model.

### An overview of nursing in India

**Fig. 1:**
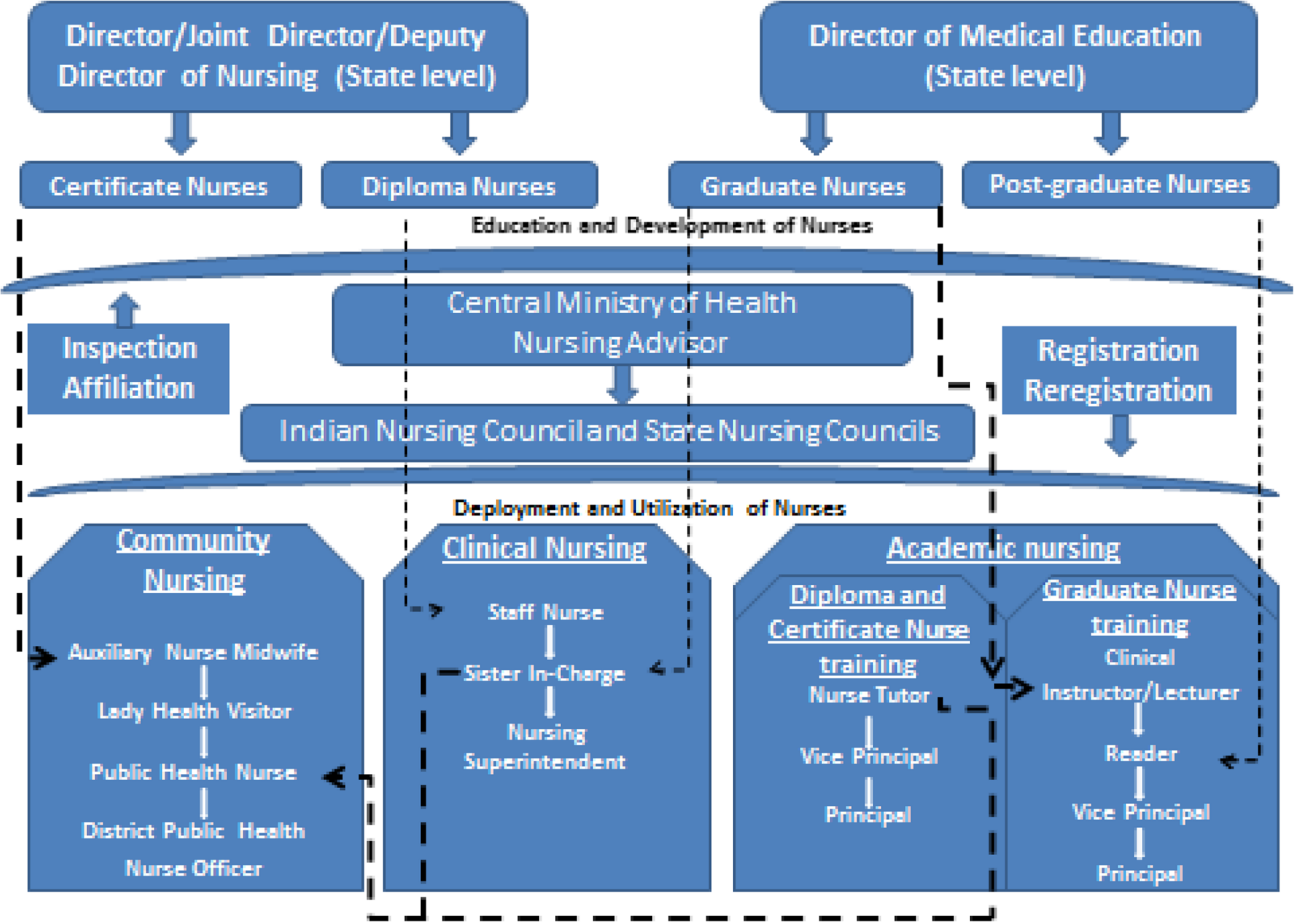
Cadre structure and functions of nurses in the health system

Figure 1 shows the hierarchical cadre structure of nurses in the Indian health system organised according to different functions.

The figure shows the complex hierarchical structure of nursing in India and the leadership positions available to nurses at the national, state and district level. There are different hierarchical structures for nursing education and administration as is the case for public health and clinical nursing. The senior most administrative position for nursing at national level is the nursing advisor in the national ministry of health. Nursing councils at the national and state levels are autonomous bodies which are mandated to regulate the nursing education and registration and monitoring of nursing practice. The different leadership positions are also designated separately for community (public health) and clinical nurses at the sub-district level. The leadership positions for community nursing such as Public Health Nurse (PHN) and District Public Health Nurse Officer (DPHNO) positions can be availed by nurses across clinical and community and education sectors as denoted by the dotted lines.

While the above structure depicts the normative requirement, there are a number of inadequacies. For example, senior positions at the level of Director or Joint Director or Deputy Director of nursing, which is the highest nursing official in provinces, are either not created or are officiated by medical doctors in most states. Similarly, a review of nursing sector in five states carried out in 2011 reported that a key public health leadership position at the district level, DPHNO is vacant in most districts [23]. The same review also identified that only very few Auxiliary Nurse Mid-wife (ANM), the entry level community nurses, are given opportunity to move to the first supervisory position of Lady Health Visitor (LHV) or higher in their entire career.

### Principal policy reform agendas for developing nurse leadership

Based on our document review, we identify the reforms that are considered crucial and grouped them under two headings, those help in uplifting the status of nursing and those which strengthen the nursing institutions, both are considered important for distributed nurse leadership.

#### Policies proposed for creating social mobility

Bhore committee report of 1946 and the High Power committee on nursing of 1987 recommended the need for nurse leaders at all levels of administration for facilitating their active participation in decision making. The pre-independence Bhore committee advocated for giving higher rank to nurses to address the low status of the Indian nursing professionals. These committees have also asked for better salary and living conditions for nurses. Another committee constituted by the government in 1954 specifically to review the service conditions of nurses recommended improvements in service and living conditions so as to attract ‘educated young women from good families’ to the profession.

Enhancing the quality of education was seen as an important step to enhance professional position of nurses among different cadres of health workers. Various committees have recommended reforming the nursing education to professionalise nursing. For example, the high power committee (1987) suggested two streams of nursing namely professional stream of graduate nurses and lesser qualified auxiliary nurses’ stream. Specialization by way of post-graduate and doctorate degrees was also proposed as an important step towards professional development [25, 26].

Improving the working conditions and providing definite paths for career progression of nurses to improve the social and professional position of nurses are two long standing demands of various nurses’ associations like Trained Nurses Association of India (TNAI) and Society of Midwifes in India [27]. The federal ministry of health and family welfare had written several letters, between 1999 to 2011 to state governments on the issue of working conditions of nurses; number of working days; allowances; upgradation of posts; payscales; promotions and study leave. TNAI’s proposal for instituting a separate professional code of conduct for nurses is another attempt to uplift the professional status and to provide distinct professional identity for nurses [27].

#### Strengthening nursing institutions

Developing nurses’ leadership relies on the capacities of key nursing institutions to deliver against their mandate and their empowerment in doing so. Different committees that looked into nursing issues have noted that nurses are generally not involved in making policies that govern their status and practice [22, 28]. The High Power committee recommended the inclusion of nurse leaders at all levels of administration in order to facilitate their active participation in planning for health sector. Institutional reforms were seen as a central component to operationalizing nurse leadership in the country. The most prominent solution put forward by the High Power committee was the establishment of Nursing Directorates at state level. With this arrangement, the committee had argued for bringing all nursing personnel technically and administratively under the control of nursing personnel themselves. The central ministry of health and family welfare has also issued guidelines in the year 2002 for establishment of separate nursing cadres in states with delegation of administrative and financial powers.

Despite several reform proposals for improving status and strengthening leadership of nurses, realization of such proposals to actual policies and its implementation remained faulty at the time of this study. A 2011 report of the central health ministry which was prepared for an expert group consultation on management capacity at the state and district level had observed this as a critical gap [29]. *“These cadres would be excluded from senior levels of management, even of their own cadre”*. Similarly, a situational analysis of status of nursing reforms in five states carried out in 2008–09 shows lack of progress in many of the reform initiatives. The Table 2 provides details on the status of nursing reforms as observed at the time of situational analysis and its implication on nursing profession and health system.

**Table 2:** Status of different policy reforms for facilitating nurse leadership based on a review of nursing sector in 5 states

Policy reforms proposed	Status of the reform	Effect of lack of action on nursing cadre and health system
Improving nurse patient ratio(Recommendation of the 1st National Health Policy 1982)	All states have made attempts to recruit additional nurses into the public health system. However, significant levels of vacancies still exist	Overburdened nurses provide poor quality nursing care
Appointment of promotional positions for public health nurses at sub-district level (Chadha Committee 1963)	Major shortfall again in filling the first promotional supervisory position in four out of five states. A higher level supervisory position of Public Health Nurse (PHN) is missing in 3 and major shortfall in 2 states	Limited promotional avenues leading to lack of motivation among nurses. Lack of professional supervision of nurses’ work
Appointment of District Public Health Nurse Officer (DPHNO). (This position was created in 1983)	Not a single post has been created in 3 states and major vacancies in 2 states	Key district level leadership for nursing sector is missing
Promotional supervisory positions for clinical nurses (A recommendation from many committees starting from Bhore Committee 1946)	Norms are not followed in creating the positions. High levels of vacancies of nursing matrons and nursing superintendents in all the states	Non recognition and no utilization of nurses in healthcare administration Low quality nursing care in hospitals
Training and skill up-gradation of in-service nurses (A recommendation from many committees starting from Bhore Committee 1946)	Sporadic training opportunities are available. Training institutions for preparing public health nurses for supervisory positions are non-functional in all states	Poor quality nursing care Less opportunity for promotions
Better working conditions for nurses including better salary and career opportunities (A recommendation from many committees starting from Bhore Committee 1946)	Grossly inadequate salary and career environment. Non-availability of proper equipment and supplies for nursing care	Poor quality nursing care
Post-graduate training opportunity for in-service nurses (A recommendation from many committees starting from Bhore Committee 1946)	No sponsored post graduate training for in-service nurses. 4 states did not have post graduate training institutions in government settings	Not enough qualified nurses available for higher positions
Creating leadership position at the state level (High power committee on nursing 1987)	2 Positions are created in one of the states, but only one position is filled. A single position was available for nurses in the state directorate in 2 states, which was not filled up due to non-availability of qualified candidates. No senior level position for nurses at the state level	Lack of direction for nursing sector in the state

### Agenda setting process for policy reforms for nurse leadership

In this section, using the three elements of Hall’s framework, we identify the inadequacies in the agenda setting process of policy reforms and try to identify the reasons and solutions based on our analysis of expert opinion.

#### Legitimacy

The process of determining the legitimacy of nurse leadership reform proposals involves examining the issues that are recognized as a problem by a significant number of policy actors especially the decision makers. Key informants interviewed for this study also noted that most of the issues identified as deficits in the nursing sector could not be resolved without the recognition of the issues by the highest levels of authority. Table 3 describes the issues which are classified as legitimate based on our analysis of key informant interviews.

**Table 3:** Legitimacy of nursing issues as perceived by the experts interviewed

Nursing policies/issues perceived to be having higher legitimacy	Nursing issues perceived to be having lesser legitimacy
• National Health Mission brought increased focus to the nurse-patient ratios, resulting in attention on staffing levels • Increased availability and reliability of data on nursing through consolidated e-registration systems • Role of nurses in the coordination and management of care in areas such as palliative and geriatric care	• Creating leadership positions for nurses at the higher levels. • Lack of capacity of nurses at managerial level • The long lead-times for application processing, candidate selection and recruitment to senior positions of nurses

In the absence of perception of severity of nurses’ leadership issue by national and state level decision makers, the legitimacy of nursing reforms is drawn towards other issues. Interviewees have noted the need for a facilitating factor, a focusing event that would unambiguously call for action and redress by government to bring attention on nursing issues back to policy makers. It was further explained that in government decision making settings, the perceived severity may be inferred from quantitative data or statistics, which allows for an appreciation of the scale of the issues at hand. Prioritization of policies for increasing nursing educational institutions and improving staffing levels in public sector is attributed by experts to the publication of large number of recent researches and data on nurse shortages and migration. The same considerations have also led to an attention on deficits in the reliability, validity and completeness of data regarding workforce quantification, qualifications, registration of nurses. The healthcare areas where nurses could add more value than medical professionals have also received recent policy attention and experts acknowledged increasing legitimacy for nurses’ leadership roles in geriatric and palliative care in recent years.

The policies supporting larger professional roles for nurses and strengthening nurse leadership were not perceived as legitimate in the sight of policy makers. Some informants saw the reason for these as embedded within the country’s socio-cultural context, particularly in relation to “woman’s place in society and her place outside the home”, which affected the perception of nursing issues by government and other stakeholders “as second class issues” (IA; IB; IF; ID; IE; IG). The dominant perception that nursing is an unskilled work, which can be equated with menial jobs is also contributing towards disregard for reforms for empowerment of nursing workforce. Some informants suggested that this lack of recognition of nursing issues is explained by insufficient clarity around what the nursing function actually entails (IG), as their role is often perceived as “limited to taking orders from doctors” (IH). The current division of labour at service delivery point was described as reflecting nursing’s subservience to the medical profession.

*Nursing profession came from UK and came as a small group to give care to British soldiers. Nursing was under a very regimented structure, trained to obey, trained to take supplementary role, not to take first role. In India there is very little chance of nursing becoming independent. (ID)*

#### Feasibility

Feasibility of policy reforms for nurse leadership is about the structural and functional capabilities required to carry the agenda of nursing leadership reforms forward. The Table 4 lists the factors identified as important for feasibility.

**Table 4:** Factors affecting the feasibility of nurse leadership according to experts interviewed

	Factors decreasing feasibility
Structural Factors	• Low levels of nursing representation at the national and provincial levels • No representational body for the profession for strongly advocating on nursing issues • Lack of clarity around authority and accountability for decision-making • Lack of role clarity between State and National level institutions as health is a “state affair”
Functional Factors	• Nurses are not appropriately prepared for leadership or managerial positions • Eligibility criteria restrict nurses from applying for leadership positions • Lack of transparency in decision-making processes • No mandate for INC for national oversight on issues other than nursing education

The experts observed that most of the issues identified as deficits in the nurse leadership sector could not be resolved without allowing nurses to hold positions of hierarchy within the government. In spite of several reform proposals, nurses’ relative position within the hierarchy of health workers has not significantly progressed over the years. “Leadership grows in social contexts and hierarchical settings, but a nurse is the lowest in this hierarchy” (ID). Informants reported that there is currently no position with decision-making power in the nursing sector and therefore, they are excluded from policy making process. “Such a post is yet to be created” (IA) and “leadership will only be achieved if we reach professional equality” (ID).

Leadership of nursing is disempowered by the high number of vacancies at key institutions, including the Ministry of Health and the Indian Nursing Council (INC). At the state level, nurses are neither involved nor represented in decision making (IG; IA; IF). Some informants stressed that the INC, the only national institution represented by nurses, may be better governed and better staffed, but they still lacked the political clout to influence decisions and policies at state level. While some informants referred to INC as the ‘go-to’ institution for nurses, others felt it neither had the constitutional authority nor the power to act upon nursing issues other than matters concerning nurses’ education (IG, IH).

Regarding the feasibility of the national directives to states (province) on strengthening of the nursing sector, the experts identified the division between state and national responsibilities for health as a factor. *“Policy direction is given to states but it is up to states to implement and there is little leverage, as the state is the supreme decision-maker in health matters*” (IC). The state nursing councils are described as weak in the state-level political hierarchy, as power is always located at the directorate of health, where nurses are neither involved nor represented. The absence of a nurse-specific institution at the state level, such as directorate of nursing is reported as a key limitation, as a separate directorate for nursing would have highlighted the issues facing the profession and advanced many policy solutions. Absence of separate nursing directorate is perceived as the most glaring gap and its establishment is considered important for allocating funding for the nursing sector and for addressing poor working conditions of nurses. *“Without it we are not represented and the powerless cannot lead”* (ID).

Improving feasibility of the proposed reform solutions to nurse leadership deficits is described as complex. Informants discussed a number of solutions, yet their feasibility is seen as hampered by a dismembered nurse leadership, corruption and a lack of vision for nursing at the institutional level. The profession is described by one of experts as *“weak in the head, led and managed by those who neither have the time or the inclination to invest their efforts at quality improvements*” (ID). The processes by which policy solutions are debated and designed were also characterized as detrimental to agenda setting. The composition of working groups and committees set up to address specific issues was seen as unrepresentative of the nursing profession (II, IB). While the inclusion of new people into such forums was seen as important, it was also recognized that high-level appointments are political and candidates are not selected on the basis of merit but rather on the degree to which they will not disrupt the status quo. *“These were systematically led by a group of tightly knit individuals” (II)*. “*This reproduces the vicious circle of inaction”*, where *“nobody wants to take the lead for fear of losing their job” (II)*.

#### Support

In line with Hall’s model, understanding the level of support for policy reforms for nurse leadership is made on the basis of experts’ opinion on nature of support from government and other decision-makers. The position of all key actors, and specifically, their non-objection to the issue were ascertained (Table 5).

Informants unanimously and unambiguously reported a lack of high level of support for developing nurse leadership at all levels of the health system. *“It is difficult to move people around this issue” (IF)*. For example, the long lead-times for application processing, candidate selection and recruitment for key nursing positions (which is described as taking on an average two years) is explained as a reflection of the low prioritization of nursing at the national level (IA).

**Table 5:** Factors affecting the support for nurse leadership among informants interviewed

Factors increasing support	Factors decreasing support
• Clear articulation of nurse leadership gaps • Right timing, with on-going high-level debates on nursing policy and governance.	• Obstruction by medical interest groups • Lack of nurse participation in decision-making

The majority of informants noted the lack of strong support from nurses themselves for these policy reforms. Lack of buy-ins from nurses and their non-participation in decision making process is described as a systemic weakness of the nursing sector. *“Leadership in nursing is dispersed across the country, disjointed in its efforts and lacked the vision, the sustenance and the unity to plead its own cause” (ID)*. A lack of belief among nurses that their conditions can be improved and their lack of interest in these matters are given as other reasons for this. The situation is described by experts with statements such as “nurses *are being against nursing” (II) or “nurses aspiring to do well… leaving the country” (ID)*.

Experts have observed that the interests of the medical profession are steering the directions of debates in health sector. Over-dominance of medical professionals is attributed to nursing profession’s disempowerment, and its lack of autonomy and independence. One respondent felt that the public opinion that shapes the perception of nursing should be challenged. *“We need to convince people about our potential” (II).* However, at the same time, securing the support of key stakeholders and making use of the current opportunities are seen as strategic. *“Beneficial alliances can be forged with the medical profession, we need to work with doctors, not against them, and if doctors take credit, then so be it. But, if it means that our profession will rise, it is worth joining hands” (II)*.

## Discussion

Policy reforms needed to address the nursing leadership crisis are challenged by the range and complexity of issues as identified in the study. The limitation of the study is the small number of key informants who participated. The Hall’s framework which analyses the process of agenda setting of policies based on the concept of legitimacy, feasibility and support provides an opportunity to compare and understand the experience of nursing leadership in other settings.

An unclear clinical responsibility assigned to nurses in their healthcare domain is described as a reason for low legitimacy for policies facilitating nurse leadership in Jordan [30]. Enhancing the image of nursing sector is seen as crucial step towards creating an enabling environment for national structures and processes that facilitate nurses’ leadership [31, 32]. The structural forces that shape the perception of nursing will be a major challenge in India. As a 1987 WHO report on nursing highlighted, “the nursing culture remains heavy with subordination without influence…(and) burdened with obligation without power” and had predicted that the nursing sector should “expect to face resistance yet take up positions from where it can voice its opinions at policy and decision-making levels” [12].

Understaffing at key national-level nursing institutions weakens the feasibility of framing relevant issues and policy solutions. The experience of nursing in other countries point to the need for creating strong leadership among academic nurses as a precursor for changes across the sector [33]. An attempt in the past to develop nurse leaders in academic setting in India has shown challenges. This isolated effort to build academic nurse leaders met with only limited and unsuitable gains in the context of several structural constrains, including social and organizational constraints [34].

Gaining support for policy reforms is contingent upon nurses’ ability to collectively demand for change. Absence of tactical advocacy strategy targeting key actors and civil society is seen as a major shortcoming of the nursing sector in India. Nurses should consider building strategic alliances across the health system to push for policy reforms promoting nurses’ leadership within the health system. Carter identifies nurses’ own reluctance to challenge the male domination of the health system as the major hurdle in changing the structural limitations of the nursing profession [35]. Fletcher argues that the style of leadership in nursing has been a reflection of an oppressed group, shaped by the oppressing social forces. He calls for increased self-reflection and dialogue as a way ahead to break the cycle of oppression and lead to changes in the structures that oppress nurses [36].

Experiences of the nursing sector in other countries resonate with Indian scenario. For example, the experience of South Africa, which is known for stronger participation of nurses in the health system, shows that nurses’ leadership development during apartheid years was strongly entangled in the political context and depended on their ability to create strategic alliances and protect self-interest [16]. Another article on Democratic Nursing Organization of South Africa describes the role played by the organization in uplifting the status of nurses by mobilising, unifying and organising nurses as a collective group in South Africa [37]. Unification of the nurses and their collective power is identified as the way forward for empowering nurses in Iran, where, as in India various contextual factors constrain nurses’ leadership potential [17, 38].

Furthermore, the possibility of achieving results with active government support should not be understated. The recent government interventions and reforms that promote scaling-up of nursing education and strengthening of nursing curricula include strong inputs towards communication, policy and planning modules [26]. The expectation is that empowered by their newly acquired skills, nurses will be able to advocate for their increased role in health sector.

## Conclusion

The absence of effective policies that create distributed and bottom-up nurse leadership in India called for this analysis of policy-making processes. This paper specifically sought to examine the agenda-setting process to understand the constraints on policy development for nurse leadership. This paper shows that the agenda of strengthening distributed nurse leadership in India is constrained by both the compromised social position of nurses and the imbalance in distribution of power and influence of nurses within the health system. This analysis also brings to attention the need to go beyond a strategy of creating few nurse leaders at the top. There is consensus among the experts that the nurse participation in decision making from the grassroots to the institutional level requires several facilitating policies targeting various levels of health system.

The study reinforces the argument that the determinants of nursing empowerment and leadership can only be addressed through deliberate attempts to enact institutional reforms that facilitate nurse leadership and through nurses’ collective advocacy to question the status quo and the structures that uphold it. This study further highlights the importance of generating further evidence on linkages between governance and policy reforms for health human resources and its influence of health system performance. A deeper understanding of the health system leadership arrangements, especially the distributed leadership will play a key role in creating equitable, efficient and accountable system.

## Abbreviations

ANM: Auxiliary Nurse Mid-wife
DPHNO: District Public Health Nurse Officer
INC: Indian Nurse Council
PHN: Public Health Nurse
TNAI: Trained Nurses Association of India
WHO: World Health Organization
